# From scroll to sale: online media engagement predicts adolescents' online supplement purchases in a nationally representative cross-sectional study

**DOI:** 10.3389/fpsyg.2026.1722369

**Published:** 2026-02-16

**Authors:** Hayriye Gulec

**Affiliations:** 1 Interdisciplinary Research Team on Internet and Society, Faculty of Social Studies, Masaryk University, Brno, Czechia; 2 Department of Psychology, Faculty of Arts and Sciences, Bursa Uludag University, Bursa, Türkiye

**Keywords:** adolescents, appearance ideals, dietary supplements, digital marketing, online information seeking, online media

## Abstract

**Background:**

Adolescents are increasingly exposed to online health information promoting supplements as quick solutions for weight management and muscle development, raising public health concerns.

**Objective:**

This study investigated whether online media engagement—weight-and fitness-related information-seeking behaviors and the internalization of online appearance ideals—predicts adolescents' online purchasing of weight-loss and muscle-building products, with attention to differences between girls and boys.

**Methods:**

A cross-sectional online survey was conducted among 1,526 Czech adolescents (50% girls) aged 13–18 years (*M* = 15.4, *SD* = 1.7). Measures included self-reported online weight- and fitness-related information-seeking behaviors, internalization of online appearance ideals (thin-ideal among girls; muscular ideal among boys), and online purchasing of weight-loss and muscle-building products. Hierarchical regression analyses were conducted separately for girls and boys, controlling for sociodemographic and body image variables.

**Results:**

Overall, 4.1% (63/1,521) purchased weight-loss and 19.1% (303/1,524) purchased muscle-building products online. For both girls and boys, greater engagement in seeking weight- and fitness-related information was associated with purchasing both product types. Internalization of online appearance ideals was significantly associated with muscle-building but not weight-loss product purchases.

**Conclusion:**

Findings highlight a novel pathway through which online media may shape youth consumer choices, pointing to the need for media literacy and body image-informed strategies to promote safer decision-making.

## 1 Introduction

Adolescents and consumers of all ages with eating disorders, body dysmorphic disorder, depression, and elevated body dissatisfaction are at heightened risk of using weight loss and muscle-building supplements, and often above manufacturer-recommended doses (Avelar-Escobar et al., 2012; Field et al., 2005; McCabe and Ricciardelli, 2009). Epidemiological studies show that unhealthy weight-control behaviors, such as diet pill use, prospectively predict increases in eating disorder symptoms and new diagnoses (Liechty and Lee, 2013; Neumark-Sztainer et al., 2006), while regular use of muscle-building supplements is linked to later steroid misuse in youth (Hildebrandt et al., 2012). Due to the evidence of harm and the vulnerability of adolescents to deceptive marketing promises, several policy recommendations have aimed to restrict the sale of these products to minors (Austin et al., 2017, 2018; Yergaliyev et al., 2020; Pomeranz et al., 2015). Yet, adolescents increasingly encounter health and appearance content online (Engel et al., 2024), where idealized images and targeted marketing may normalize supplement use and facilitate purchasing (Buchanan et al., 2018; Lacko et al., 2024). This environment may amplify previously documented risks, but no studies have examined how adolescents' online media engagement translates into their online purchasing of supplements. To address this gap, the present study investigates whether the internalization of online appearance ideals and online weight- and fitness-related information-seeking behaviors predict online purchasing of weight-loss and muscle-building products.

The weight loss and muscle-building supplement market is large and readily accessible to young people (Svantorp-Tveiten et al., 2021). In a study of more than 10,000 adolescents, approximately 4.7% of boys and 1.6% of girls reported using protein powder or shakes, creatine, amino acids/HMB, dehydroepiandrosterone, growth hormone, or anabolic/injectable steroids at least weekly (Field et al., 2005). Other work found that 68% of youth were potential supplement users and nearly half believed that protein supplements enhanced performance, despite insufficient evidence for recreational users (Bell et al., 2004). Adolescents also reported perceived physiological benefits and limited awareness of risks (O'Dea, 2003), beliefs often shaped by unmonitored online information (Ruano and Teixeira, 2020), accessed via retailer websites, targeted advertisements, and fitness-related platforms. Indeed, online sources are the most frequently used channels to learn about these products (Ruano and Teixeira, 2020; Ganson et al., 2024).

The Tripartite Influence Model identifies media, peers, and parents as key sociocultural influences on body image, disordered eating, and appearance-related behaviors (Thompson et al., 1999a,b; van den Berg et al., 2002). Guided by this model, research indicates that internalizing ideal media appearances is associated with thinness and muscularity-oriented behaviors in adolescents (van den Berg et al., 2002). In the context of supplements, the search for an ideal body and enhancing appearance or athletic performance are common motivations (Alves and Lima, 2009; Barretto et al., 2024), and social media exposure and influencer engagement have emerged as significant contributors to adolescents‘ use of appearance and performance-enhancing substances (Ganson et al., 2023, 2024). However, whether internalization of idealized online appearances—a central mechanism in the model—extends to adolescents' online supplement purchasing remains unexamined, despite its relevance for policy and prevention efforts targeting digital marketing practices and appearance pressures.

Prior research also indicates that adolescents who use supplements are more likely to seek online weight- and fitness-related information, and that such information-seeking predicts the use of a broader array of appearance- and performance-enhancing substances, rather than merely the time spent on social media (Ganson et al., 2023, 2024). These patterns align with Uses and Gratifications Theory (Ruggiero, 2000), which posits that individuals engage with media to fulfill specific needs; in this case, adolescents may intentionally seek out content that promotes substances promising enhanced appearance or performance. This reliance on online information—often commercialized, unregulated, or misleading (Almenara and Gulec, 2024)—is especially concerning during adolescence, a sensitive developmental period for body image (Vankerckhoven et al., 2023) and heightened susceptibility to deceptive claims (Greškovičová et al., 2022; Lacko et al., 2024). Yet, it remains unknown whether such information-seeking behaviors translate into online purchasing of supplements.

Therefore, this study examines whether internalizing online appearance ideals and seeking online weight- and fitness-related information predict adolescents' online purchases of weight loss and muscle-building products in a nationally representative sample of Czech adolescents. Given prior findings linking gender-related norms and appearance expectations to weight control behaviors (Nagata et al., 2020), the analyses consider boys and girls separately and distinguish between purchases of weight loss and muscle-building products. The internalization of online appearance ideals was assessed in terms of the thin ideal among girls and the muscular ideal among boys. The supplement types enquired about the frequency of purchase attempts to lose weight and gain muscle, regardless of the product purchased. The analyses controlled for age and socioeconomic status, which are associated with supplement use (Hoffman et al., 2008; Bell et al., 2004; Picciano et al., 2007), as well as the time adolescents spent online (Tiggemann and Slater, 2014), body mass index (BMI), and body dissatisfaction (Nagata et al., 2019; Picciano et al., 2007).

In summary, despite evidence linking online media exposure, influencer engagement, and information-seeking to supplement use (Ganson et al., 2023, 2024), it remains unclear whether these processes extend to online purchasing—the behavioral endpoint that directly exposes adolescents to potentially harmful products. The present study addresses this gap by examining whether internalization of online appearance ideals and online weight- and fitness-related information-seeking are associated with online purchasing of weight loss and muscle-building supplements. Understanding this pathway provides insight into how digital environments may reinforce risky consumer behaviors, informing public health and regulatory strategies aimed at protecting adolescent well-being.

## 2 Materials and methods

### 2.1 Participants and procedure

This study is part of a larger project that sampled adolescents and one of their parents. The target group for eligibility consisted of Czech households with at least one parent or caregiver and at least one adolescent aged 13–18 years who were able to complete the online questionnaire. From each eligible household, only one parent/caregiver and one adolescent were included in the study. Thus, in households with multiple adolescents or caregivers, a single parent/caregiver–adolescent dyad was selected for participation. The present study includes all adolescents who met the eligibility and quality-control criteria of the project.

Data collection was conducted by a professional research agency that is a member of the European Society for Opinion and Market Research (ESOMAR) and adheres to its standards for ethical and professional conduct. Participants were recruited from the agency's online panel. Quota sampling was employed to ensure a balanced distribution across girls and boys, and age, and to achieve proportional representation of Czech households with children, based on household income, administrative region (as defined by the Nomenclature of Territorial Units for Statistics), and municipality size. The agency selected eligible participants for the final sample from an initial pool of 12,664 adolescent–parent dyads. Of this pool, 60% did not open the survey, 14% opened it but did not provide any responses, and 2% failed to answer the initial screening questions. An additional 12% were excluded due to quota constraints on sampling. Because the study was embedded within the national project, the sample size was determined by representativeness requirements rather than a separate a priori power analysis. Nonetheless, a post-hoc power analysis provided excellent statistical power to detect small incremental effects (f^2^ = 0.02–0.04) in the planned analyses.

The study employed the CAWI (Computer-Assisted Web Interviewing) method. Adolescents and their parents completed an online questionnaire from their homes, with only one adolescent and one parent recruited per household. The agency invited its panelists to participate in the study via e-mail, providing details about the survey, eligibility criteria, and instructions for completing the questionnaire. The invitation included a link to the adolescent questionnaire, allowing parents to review the content beforehand. After submitting the basic information required for quota sampling and giving active consent for their child's participation, parents were instructed to invite the adolescent to complete the questionnaire. They were also encouraged to ensure the adolescent's privacy during survey completion. At the beginning of the questionnaire, adolescents were asked for their active consent and informed about the survey's purpose, confidentiality, and their right to decline participation. They were also aware that they could respond to any question with “I don't know” or “I prefer not to say.” The Research Ethics Committee of Masaryk University approved the study.

The survey was thoroughly pretested through cognitive interviews (i.e., 30 adolescents and two parents) and a pilot study (i.e., 195 adolescent-caregiver dyads) to ensure the comprehension of the questionnaires, check the data distributions, and determine the dimensions and internal reliabilities of the scales. Quality-control procedures were applied throughout data collection: the agency monitored questionnaire completion times, assessed the consistency of entries between parent and adolescent respondents, and removed participants whose responses failed quality checks or who completed less than 90% of the questionnaire. Approximately 1% of respondents were removed for these reasons. The final sample consisted of 1,526 adolescents (764/1,526, 50.01% girls) aged 13–18 years (M = 15.4, SD = 1.7).

### 2.2 Measures

#### 2.2.1 Internalization of online body-ideal content

The internalization of online body-ideal content was assessed using an adapted version of the Internalization Subscale from the Multidimensional Media Influence Scale (Cusumano and Thompson, 2001). The original scale was developed to measure media-related (TV, magazines) influences on body image in children and preadolescents, with subscales evaluating awareness of media ideals, perceived pressure from media, and internalization of media ideals. The scale demonstrated adequate psychometric properties, with internal consistency coefficients ranging from 0.68 to 0.92. For the present study, four items representing the Internalization subscale were selected and adapted to specifically reflect adolescents' exposure to online content that idealizes appearance. The items inquired about the extent to which adolescents tended to internalize ideal appearances commonly presented in online photos and videos, with the following questions: “I compare my figure with theirs,” “I try to look like them,” “They inspire me in how to look attractive,” “I would like my figure to be the same as theirs is.” Girls responded by considering the photos or videos of girls/women that portrayed the “feminine beauty ideal” (i.e., skinny figure) on Instagram or elsewhere on the internet. Boys considered the photos or videos of boys/men portraying the “masculine beauty ideal” (i.e., muscular and toned figure). Responses were provided on a 5-point Likert scale (1 = very untrue of me to 5 = very true of me). Higher scores reflected greater internalization of online appearance ideals. The adapted subscale demonstrated high internal consistency in the current sample (Cronbach's α = 0.88).

#### 2.2.2 Online weight- and fitness-related information seeking

Adolescents reported how often they had used the internet in the past few months to search for information, discussions, articles, or posts related to “healthy eating and nutrition,” “dietary supplements or vitamins,” “how to exercise and do sports (non-professional),” and “losing weight (e.g., diets and weight loss tutorials).” Items were rated on a 6-point scale ranging from 1 = never to 6 = several times a day, with higher scores indicating more frequent weight- and fitness-related online information seeking. The items were developed by the project team and derived from prior research on adolescents‘ online health information-seeking behaviors (Wartella et al., 2016; Rideout and Fox, 2018; Rideout et al., 2021). Content validity was further supported through expert review by researchers specializing in adolescent health and digital behavior. The measure uses direct behavioral frequency items, a format widely employed in studies of adolescents' online health information seeking and digital health behaviors (Saraipour et al., 2025). In the present sample, the scale demonstrated good internal consistency (Cronbach's α = 0.83).

#### 2.2.3 Online purchasing of weight loss and muscle-building products

Adolescents answered two separate questions inquiring about how often they had bought products to lose weight and gain muscle online in the past year to assess their online purchasing behaviors. The response options included “never” (1), “a few times at most” (2), “several times a month” (3), and “several times a week” (4).

#### 2.2.4 Control variables

The participants indicated their age in years and their sex, using a binary response format: “girl” (1) and “boy”(2). The BMI was calculated based on adolescents' self-reported responses to open-ended questions regarding their height (in centimeters) and weight (in kilograms). Time spent online was assessed with the following question: “About how much time do you spend on the internet during a typical weekday (Monday–Friday)?” The items were evaluated on a 9-point Likert scale with response options ranging from little or no time (1) to about 7 hours or more (9). Parental reports were used to evaluate socioeconomic status. The question was, “How can your household make ends meet on your monthly income?” The response options ranged from “great difficulty” (1) to “very easily” (6). In addition to the single-item indicator of parental perceived financial strain, socioeconomic diversity was ensured at the sampling level, as quota sampling required the distribution of household income in the sample to match that of Czech households with children (with a tolerance of ±10%).

Body dissatisfaction was measured using five positively phrased items adapted from the Body Dissatisfaction subscale of the Eating Disorder Inventory-3 (Garner, 2004). The adapted five-item set—covering satisfaction with the overall figure, thighs, stomach, hips, and buttocks—follows the approach used in a recent Czech adolescent study that included the present sample, along with additional participants (Kvardova et al., 2023). In that study, the items demonstrated acceptable internal consistency and supported a unidimensional structure for the Body Dissatisfaction subscale. Adolescents indicated how true each statement was for them on a five-point scale ranging from (1) “very untrue of me” to (5) “very true of me,” with lower scores indicating greater body dissatisfaction. Consistent with previous Czech findings, internal consistency in the present sample was satisfactory (Cronbach's α = 0.79).

### 2.3 Statistical analysis

Descriptive statistics were run for the sociodemographic characteristics (age, sex, socioeconomic status), BMI, body dissatisfaction, and the study variables. Differences between girls and boys were tested using the independent samples *t*-test. Two separate two-stage multiple hierarchical regression analyses were conducted, with online purchasing of weight loss and muscle-building products as the dependent variables. Age, socioeconomic status, time spent online, BMI, and body dissatisfaction were the control variables entered at the first stage of the analyses. Variables concerning adolescents' online weight- and fitness-related information-seeking behaviors and internalization of online body ideals were entered at the second stage. The analyses were conducted separately for girls and boys. Collinearity and multivariate outliers were examined before the analyses. The collinearity statistics revealed that tolerance and variance inflation factor (VIF) statistics were within the acceptable limits for the independent variables (tolerance values were above 0.2 and VIF values were less than 4). An examination of the Mahalanobis distance values indicated four multivariate outliers, which were deleted from the dataset. The analyses were run using the Statistical Package for Social Sciences (SPSS) version 28 (IBM Corp, 2021). A two-tailed α = 0.05 was applied to statistical testing.

## 3 Results

### 3.1 Sample characteristics

The sample comprised 1,526 adolescents (764/1,526, 50.01% girls). Of the total sample, 4.1% (63/1,521) had ever purchased weight loss products online [girls: 5.2% (40/763), boys: 3% (23/758)]. Muscle-building products were purchased more frequently. Of the total sample, 19.9% (303/1,524) reported purchasing muscle-building products online [girls: 19.5% (149/764), boys: 20.3% (154/760)].

Table 1 represents the characteristics of the sample and the differences by sex. The mean age of participants was 15.37 (*SD* = 1.71). The total monthly income of the households could make ends meet between “with some difficulty” and “quite easily.” The average time adolescents spent using the internet approximated 5 h a day on typical weekdays. There were significant differences between girls and boys in internet use [*t* (1507) = −2.402, *p* = 0.016], body dissatisfaction [*t* (1450) = −4.907, *p* = < 0.001], internalization of online appearance ideals [*t* (1502) = 8.993, *p* = < 0.001] and online weight- and fitness-related information seeking behaviors [*t* (1516) = 8.015, *p* = < 0.001]. Boys spent more time using the internet. On the other hand, girls were using the internet more frequently to seek information related to weight and fitness. They were also more likely than boys to report higher body dissatisfaction and internalization of online beauty ideals. No significant differences were observed in the mean frequency of weight loss and muscle-building product purchases across girls and boys.

**Table 1:** Sociodemographic characteristics of the whole sample and by sex (girls and boys) (*N* = 1,526).

**Variables**	**Total sample (*N* = 1,526)**	**Girls (*N* = 764)**	**Boys (*N* = 762)**	***p***
	**Mean (*SD*)**	**Mean (*SD*)**	**Mean (*SD*)**	
Age (in years)	15.37 (1.71)	15.38 (1.68)	15.37 (1.74)	0.91
Socioeconomic status	3.67 (1.07)	3.66 (1.05)	3.68 (1.1)	0.81
Time spent online	6.61 (1.83)	6.49 (1.82)	6.72 (1.83)	**0.02**
BMI (kg/m^2^)	21.09 (3.69)	20.96 (3.62)	21.2 (3.76)	0.25
Body dissatisfaction	3.28 (0.89)	3.17 (0.92)	3.39 (0.84)	**< 0.001**
Internalization	2.77 (1.13)	3.02 (1.1)	2.51 (1.11)	**< 0.001**
Online weight-and fitness-related information seeking	2.27 (0.98)	2.47 (0.99)	2.07 (0.93)	**< 0.001**
Online purchasing of weight loss products	1.06 (0.33)	1.08 (0.35)	1.05 (0.31)	0.11
Online purchasing of muscle-building products	1 (0.6)	1.25 (0.56)	1.29 (0.64)	0.21

BMI, body mass index. Parental reports were used to assess socioeconomic status, with response options ranging from “great difficulty” (1) to “very easily” (6). Time spent online represents adolescent-reported hours spent online on a typical weekday, assessed on a 9-point scale ranging from “little or no time” (1) to “about 7 h or more” (9). Body dissatisfaction was assessed using an adapted version of the Body Dissatisfaction subscale from the Eating Disorder Inventory−3. Internalization assessed online thin ideal for girls and online muscular ideal for boys with an adapted version of the Internalization subscale from the Multidimensional Media Influence Scale. Response options ranged from “very untrue of me” (1) to “very true of me” (5) for body dissatisfaction and internalization; from “never” (1) to “several times a day” (6) for online weight-and fitness-related information seeking; and from “never” (1) to “several times a week” (4) for online purchasing of weight loss and muscle-building products.

Significant *p* values are shown in boldface, *p* < 0.05.

### 3.2 Predictors of online purchasing of weight loss products

The hierarchical multiple regression analysis to predict online purchasing of weight loss products among girls at Stage 1 revealed that control variables (age, SES, time spent online, BMI, body dissatisfaction) contributed significantly to the model (*R*^2^ = 0.03, *F* (5, 634) = 3.96, *p* = 0.002). Introducing variables related to the internalization of online thin beauty ideals and the frequency of online weight- and fitness-related information-seeking behaviors was associated with a significant additional 9% variation in Stage 2 [*F* (2, 632) = 31.02, *p* < 0.001]. The final model was statistically significant [*R*^2^ = 0.12, *F* (7, 632) = 11.96, *p* < 0.001]. The results demonstrated that adolescent girls with a higher BMI were more likely to purchase online weight loss products in Stage 1 of the analyses. In Stage 2, the predictors of online purchasing of weight loss products were a higher BMI and a higher frequency of online weight- and fitness-related information seeking behaviors.

For boys, the control variables (age, SES, time spent online, BMI, body dissatisfaction) did not predict online purchasing of weight loss products [*R*^2^ = 0.01, *F* (5, 619) = 1.13, *p* = 0.35]. Introducing variables related to the internalization of online muscular ideals and frequency of online weight- and fitness-related information seeking behaviors was associated with a significant additional 9% variation in Stage 2 [*F* (2, 617) = 30.27, *p* < 0.001]. The final model was statistically significant [*R*^2^ = 0.1, *F* (7, 617) = 9.53, *p* < 0.001]. The results demonstrated that SES and the frequency of online weight- and fitness-related information-seeking behaviors were significant predictors of online purchasing of weight loss products in Stage 2 of the analyses. Adolescent boys with a better socioeconomic status and those who engaged with online weight- and fitness-related information seeking behaviors at a higher frequency were more likely to purchase them. The summary of the findings for predicting online purchasing of weight loss products for girls is shown in Table 2, and for boys in Table 3. It should be noted that the final models explained 12% of the variance in girls and 10% in boys.

**Table 2:** Summary of the multiple hierarchical regression analysis predicting online purchasing of weight loss products in girls (*N* = 640).

**Variables**	**Model 1** ^**a**^			**Model 2** ^**b**^		
	***b* (95% CI)**	**β**	***p***	***b* (95% CI)**	**β**	***p***
**Block 1**						
Age	0.01 (−0.01; 0.03)	0.05	0.21	−0.01 (−0.02; 0.01)	−0.03	0.49
Socioeconomic status	0.01 (−0.02; 0.03)	0.02	0.63	0.00 (−0.02; 0.03)	0.01	0.88
Time spent online	0.00 (−0.01; 0.02)	0.01	0.87	0.00 (−0.01; 0.02)	0.00	0.91
BMI	0.02 (0.01; 0.02)	0.15	**< 0.001**	0.02 (0.01; 0.02)	0.15	**< 0.001**
Body dissatisfaction	−0.00 (−0.04; 0.03)	−0.01	0.83	0.00 (−0.03; 0.04)	0.02	0.72
**Block 2**						
Internalization				0.02 (−0.01; 0.04)	0.05	0.26
Online weight-and fitness-related information seeking				0.10 (0.07; 0.13)	0.29	**< 0.001**

BMI, Body Mass Index. Variable definitions and measurement details are provided in Table 1.

^a^R^2^ = 0.03, adjusted R^2^ = 0.02.

^b^R^2^ = 0.12, adjusted R^2^ = 0.11, ΔR^2^ = 0.09, *p* < 0.001.

Significant *p* values are shown in boldface.

**Table 3:** Summary of the multiple hierarchical regression analysis predicting online purchasing of weight loss products in boys (*N* = 625).

**Variables**	**Model 1** ^**a**^			**Model 2** ^**b**^		
	***b* (95% CI)**	**β**	***p***	***b* (95% CI)**	**β**	***p***
**Block 1**						
Age	−0.00 (−0.02; 0.01)	−0.01	0.83	−0.01 (−0.02; 0.01)	−0.05	0.26
Socioeconomic status	0.02 (−0.00; 0.04)	0.07	0.11	0.02 (0.00; 0.05)	0.08	**0.03**
Time spent online	0.0 (−0.01; 0.01)	0.00	0.98	0.00 (−0.01; 0.01)	0.00	0.91
BMI	0.00 (−0.00; 0.01)	0.04	0.34	0.00 (−0.01; 0.01)	0.02	0.59
Body dissatisfaction	−0.02 (−0.05; 0.01)	−0.06	0.2	−0.02 (−0.05; 0.01)	−0.07	0.11
**Block 2**						
Internalization				−0.02 (−0.04; 0.01)	−0.05	0.2
Online weight-and fitness-related information seeking				0.10 (0.08; 0.13)	0.32	**< 0.001**

BMI, Body Mass Index. Variable definitions and measurement details are provided in Table 1.

^a^R^2^ = 0.01, adjusted R^2^ = 0.00.

^b^R^2^ = 0.1, adjusted R^2^ = 0.09, ΔR^2^ = 0.09, *p* < 0.001.

Significant *p* values are shown in boldface.

### 3.3 Predictors of online purchasing of muscle-building products

The hierarchical multiple regression analysis to predict online purchasing of muscle-building products among girls at Stage 1 revealed that control variables (age, SES, time spent online, BMI, and body dissatisfaction) contributed significantly to the model [*R*^2^ = 0.06, *F* (5, 634) = 7.77, *p* < 0.001]. Introducing variables related to the internalization of online thin beauty ideals and the frequency of online weight- and fitness-related information-seeking behaviors was associated with a significant additional 15% variation in Stage 2 [*F* (2, 632) = 59.29, *p* < 0.001]. The final model was statistically significant [*R*^2^ = 0.21, *F* (7, 632) = 25.51, *p* < 0.001]. The results demonstrated that older age was a significant predictor of online purchasing of muscle-building products in Stage 1 of the analyses. In Stage 2, the predictors included age, internalization of thin beauty ideals, and frequency of online weight- and fitness-related information-seeking behaviors. Older adolescent girls, and those with higher scores on internalizing online thin beauty ideals and who searched the internet more frequently for weight- and fitness-related information, were more likely to purchase muscle-building products online.

For boys, the control variables (age, SES, time spent online, BMI, and body dissatisfaction) contributed significantly to the model predicting online purchasing of muscle-building products [*R*^2^ = 0.04, *F* (5, 620) = 4.49, *p* = 0.001]. Introducing variables related to the internalization of online muscular ideals and the frequency of online weight- and fitness-related information-seeking behaviors was associated with a significant additional 31% variation in Stage 2 [*F* (2, 618) = 148.11, *p* < 0.001]. The final model was statistically significant [*R*^2^ = 0.35, *F* (7, 618) = 47.04, *p* < 0.001]. Similar to girls, older age was a significant predictor of online purchasing of muscle-building products in both stages. Also, adolescent boys with higher scores on internalizing online muscular ideals and who searched the internet more frequently for weight- and fitness-related information were more likely to purchase muscle-building products online. The summary of the findings for predicting online purchasing of muscle-building products for girls is shown in Table 4, and for boys in Table 5. It is worth noting that the final models explained 21% of the variance in girls and 35% in boys.

**Table 4:** Summary of the multiple hierarchical regression analysis predicting online purchasing of muscle-building products in girls (*N* = 640).

**Variables**	**Model 1** ^**a**^			**Model 2** ^**b**^		
	***b* (95% CI)**	**β**	***p***	***b* (95% CI)**	**β**	***p***
**Block 1**						
Age	0.07 (0.04; 0.1)	0.21	**< 0.001**	0.04 (0.01; 0.06)	0.11	**0.006**
Socioeconomic status	0.04 (−0.00; 0.08)	0.07	0.08	0.03 (−0.01; 0.07)	0.05	0.16
Time spent online	0.00 (−0.02; 0.03)	0.00	0.92	0.00 (−0.02; 0.02)	0.00	0.97
BMI	0.01 (−0.01; 0.02)	0.05	0.26	0.01 (−0.01; 0.02)	0.04	0.31
Body dissatisfaction	−0.03 (−0.08; 0.03)	−0.04	0.31	−0.00 (−0.05; 0.05)	−0.01	0.89
**Block 2**						
Internalization				0.04 (0.00; 0.09)	0.08	**0.043**
Online weight-and fitness-related information seeking				0.21 (0.17; 0.26)	0.36	**< 0.001**

BMI, Body Mass Index. Variable definitions and measurement details are provided in Table 1.

^a^R^2^ = 0.06, adjusted R^2^ = 0.05.

^b^R^2^ = 0.21, adjusted R^2^ = 0.20, ΔR^2^ = 0.15, *p* < 0.001.

Significant *p* values are shown in boldface.

**Table 5:** Summary of the multiple hierarchical regression analysis predicting online purchasing of muscle-building products in boys (*N* = 626).

**Variables**	**Model 1** ^**a**^			**Model 2** ^**b**^		
	***b* (95% CI)**	**β**	***p***	***b* (95% CI)**	**β**	***p***
**Block 1**						
Age	0.06 (0.03; 0.09)	0.16	**< 0.001**	0.03 (0.01; 0.06)	0.09	**0.007**
Socioeconomic status	0.00 (−0.04; 0.05)	0.01	0.87	0.03 (−0.01; 0.07)	0.05	0.15
Time spent online	−0.00 (−0.04; 0.02)	−0.02	0.63	−0.01 (−0.03; 0.02)	−0.02	0.58
BMI	0.01 (−0.01; 0.03)	0.06	0.18	0.01 (−0.01; 0.02)	0.03	0.37
Body dissatisfaction	0.04 (−0.02; 0.11)	0.06	0.19	0.04 (−0.01; 0.09)	0.05	0.14
**Block 2**						
Internalization				0.07 (0.03; 0.11)	0.12	**0.001**
Online weight-and fitness-related information seeking				0.35 (0.30; 0.4)	0.51	**< 0.001**

BMI, Body Mass Index. Variable definitions and measurement details are provided in Table 1.

^a^R^2^ = 0.04, adjusted R^2^ = 0.03.

^b^R^2^ = 0.35, adjusted R^2^ = 0.34, ΔR^2^ = 0.31, *p* < 0.001.

Significant *p* values are shown in boldface.

## 4 Discussion

This study presents the first comprehensive information on the role of online media in adolescents‘ purchases of weight loss and muscle-building products over the internet. Adolescents' self-reported internalization of online appearance ideals and weight- and fitness-related information-seeking behaviors were evaluated as predictors. The analyses were conducted separately for girls and boys, controlling for sociodemographic and body image-related characteristics. The results elucidated distinct patterns for girls and boys in online supplement purchases for weight loss and muscle-building supplements in a nationally representative sample of Czech adolescents.

Regardless of whether adolescents were girls or boys, a higher frequency of seeking online weight- and fitness-related information was associated with purchasing weight loss and muscle-building products online. Previous research has shown an association between using products to improve appearance or strength and visiting fitness websites and reading health/fitness magazines in adolescent boys (Frison et al., 2013; Field et al., 2005). The present study extends these previous findings by indicating an association between seeking online weight- and fitness-related information and online purchasing of weight loss and muscle-building products across both girls and boys. One possible interpretation of these findings concerns exposure. Adolescents who frequently seek online weight- and fitness-related information might be exposed to fitness-related product ads on these websites, encouraging their purchase. On the other hand, it is also possible that purchasing fitness products and seeking weight- and fitness-related information online might serve as lifestyle behaviors among those who use supplements. Although the cross-sectional design of this study limits the ability to determine the directionality of these associations, it is plausible to assume a reciprocal association where both interpretations might mutually reinforce each other, with significant public health implications.

Adolescents frequently report that online websites are the primary source of information from which they learn about supplements (Ruano and Teixeira, 2020; Ganson et al., 2024). Previous research has shown that seeking online weight- and fitness-related information is associated with the use of a variety of supplements in adolescents, compared to merely spending time on social media (Ganson et al., 2023, 2024). By exposing adolescents to a plethora of information and advertisements on how to lose weight and improve athletic performance, these websites could serve as a gateway for consumerism of supplements, offering fast and easy solutions. For those who already use supplements, weight- and fitness-related websites may create a self-sustaining fitness ecosystem that influences purchasing behaviors, most likely due to the reinforcement of appearance-related goals, exposure to persuasive marketing messages, and the normalization of supplement use as a quick and accessible solution for achieving idealized body standards. This dynamic may perpetuate ongoing purchasing behaviors and increase adolescents' vulnerability to misinformation, risky consumption patterns, and long-term health consequences, underscoring the need for regulatory oversight and media literacy interventions from a public health perspective.

The present findings indicated that adolescents who internalized culturally promoted online appearance ideals—specifically, the thin ideal for girls and the muscular ideal for boys—were significantly more likely to purchase muscle-building supplements online. This finding suggests that when young people adopt these appearance standards as personal goals or values, it can shape their consumer behavior in digital spaces. While previous research has already shown that internalizing appearance ideals can lead to harmful outcomes such as body dissatisfaction, eating disorders, and other risky health behaviors (e.g., engaging in extreme dieting, excessive exercise, and muscle building) (Rodgers et al., 2020; Levine and Murnen, 2009), this study makes a novel contribution by linking such internalization specifically to the online purchasing of muscle-building products. These findings align with the Tripartite Influence Model, which posits that sociocultural agents, particularly media, influence how adolescents perceive and manage their appearance (Thompson et al., 1999a,b; van den Berg et al., 2002). The present study advances this model by demonstrating its applicability to adolescents' appearance-oriented digital consumerism, underscoring the commercial implications of internalized media ideals within contemporary online environments.

It should be noted, however, that adolescents' gender identities and sexual orientations extend beyond the binary sex classification (girls/boys) employed in the present study, and prior research suggests that these aspects of identity are associated with differences in body-related attitudes and behaviors. For example, sexual minority boys have been shown to report greater preoccupation with leanness compared to their heterosexual peers (Calzo et al., 2015), whereas lesbian and bisexual girls tend to express higher body satisfaction and report less pressure to conform to media-driven appearance ideals than heterosexual girls (Austin et al., 2004). More recent evidence indicates that these patterns are not uniform, as sexual minority women have been found to experience elevated muscle dissatisfaction compared to heterosexual women (Henriksen et al., 2025), and gay men have reported significantly higher engagement in muscularity-oriented eating behaviors than heterosexual men (Lev Arey et al., 2025). Research on gender-expansive individuals further suggests that appearance-related pressures may be shaped by experiences of fatphobia, a strong thinness orientation, and tensions arising from nonconformity to dominant gender norms related to femininity (Biefeld et al., 2025). In addition, a growing cultural emphasis on strength, athleticism, and physical functionality among girls—often reinforced through sport participation and fitness-oriented media—may contribute to the adoption of muscularity-oriented appearance goals independent of sexual orientation or gender identity (Bozsik et al., 2018). Taken together, these findings suggest that the purchasing of muscle-building supplements by both boys and girls may reflect not only traditional gender norms but also alternative appearance ideals related to strength, empowerment, or community-specific beauty standards.

In contrast, the association between internalization of online appearance ideals and online purchasing behavior did not extend to weight loss products. Among both boys and girls, no statistically significant relationship was found between the internalization of online appearance ideals and the frequency of purchasing weight loss supplements online. This finding suggests that the psychological mechanisms driving adolescents to buy muscle-building supplements may differ from those influencing the purchase of weight loss products. The absence of a significant association for either girls or boys indicates that internalizing thinness or muscularity ideals—despite being conceptually tied to weight control (Nagata et al., 2020)—may not directly translate to consumer behaviors aimed at weight loss in the same way they do for muscle gain. Therefore, it is recommended to distinguish between various categories of appearance-related products when examining the behavioral outcomes of media engagement. Future research could determine whether factors, such as parental control, price sensitivity, product accessibility, or stigma surrounding weight loss supplements, may mediate or inhibit such purchasing behavior among adolescents, despite internalized ideals.

Regarding the control variables, girls with a higher BMI were more likely to purchase online weight loss products. This finding is consistent with prior research indicating an association between a higher BMI and unhealthy weight control behaviors in adolescent girls (Boutelle et al., 2002). It highlights that a similar association might extend to online consumer behaviors for weight loss products. For boys, none of the control variables was associated with online purchasing of weight loss products in the first stage of the analysis, and the model was insignificant. However, when variables related to media engagement—internalization of muscular ideal and online weight- and fitness-related information-seeking behaviors—were entered into the model, the results revealed that boys with a higher socioeconomic status were more likely to purchase weight loss products online. The increased model fit in the second stage could partly explain this finding. Although socioeconomic status was not a significant predictor when the model fit was poor, including variables related to media exposure might have provided the context to increase its significance in the second stage. It is also possible that higher socioeconomic status confers greater access to online marketplaces and discretionary spending, which, when combined with media-driven appearance pressures, may facilitate engagement in appearance-oriented consumption behaviors.

Among both girls and boys, age was a significant predictor of online purchasing of muscle-building products. Reliance on nutritional supplements to alter body appearance increases as adolescents mature (Hoffman et al., 2008). The present findings suggest that this tendency could extend to online purchasing of muscle-building products. Older adolescents may experience increased peer comparison and social evaluation related to appearance, which has been linked to elevated body image concerns during adolescence (Helfert and Warschburger, 2011). In addition, age-related increases in autonomy, financial independence, and unsupervised internet access may lower barriers to purchasing supplements online (Subrahmanyam and Šmahel, 2011). Muscle-building products may also be perceived as tools for health, attractiveness, or weight management, given widespread beliefs that increased muscle mass enhances metabolism and appearance (O'Dea, 2003; Yanover and Thompson, 2010). It should be noted that the analyses did not distinguish the types of muscle-building products purchased; thus, the possibility of acquiring illegal or unsafe substances cannot be ruled out. Such behaviors may normalize risky consumption patterns, reinforce unrealistic body ideals, and heighten vulnerability to disordered eating. Accordingly, targeted media literacy interventions and regulatory measures addressing digital consumerism should consider age-related factors in adolescents' online purchasing of muscle-building products.

### 4.1 Limitations and future research directions

The findings of the present study should be interpreted with several limitations in mind. First, the study relied on self-report measures, which may be subject to response biases. Second, the cross-sectional design limits the ability to draw conclusions. Also, socioeconomic status was assessed using perceived household financial strain reported by parents rather than a multi-item socioeconomic index. While the survey's quota-based sampling helped ensure representation across the socioeconomic spectrum, the use of a single-item indicator may still limit measurement precision.

Third, online weight- and fitness-related information-seeking was measured without specifying the exact sources or platforms used (e.g., supplement-focused websites vs. general health websites), which reduced the interpretability of the digital environments involved. Similarly, overall online screen time captures total time spent online but not the proportion devoted to appearance-, weight-, or fitness-related content. This lack of specificity may have influenced the magnitude of associations observed in the hierarchical model. The analyses also did not control for adolescents' engagement in sports or fitness activities, which are known correlates of supplement use (Field et al., 2005). In addition, appearance-ideal internalization was operationalized using thinness for girls and muscularity for boys (Nagata et al., 2020); however, emerging work highlights the relevance of the fit-ideal (Roberts et al., 2022). The explanatory power of the models was modest, particularly for online purchasing of weight-loss products (12% in girls and 10% in boys), compared with stronger effects for muscle-building products (21% in girls and 35% in boys).

Further, potentially relevant confounders—such as e-health literacy, eating disorder symptoms, or adolescents' involvement in online work—were not assessed. Because the study did not distinguish between legal over-the-counter supplements and illicit/pharmacologically active weight-loss or muscle-building products, the health risk implications remain unclear. Previous research shows that many products marketed for weight loss or muscle building are adulterated with undeclared synthetic agents, thereby increasing the potential harm (Rocha et al., 2016). Finally, reports of online purchasing behaviors may not fully reflect actual consumption patterns. Therefore, caution is warranted when drawing parallels between purchasing and actual use.

Importantly, the study did not assess adolescents' emotional responses to purchasing supplements (e.g., satisfaction, regret, or perceived pressure), nor their perceptions of product effectiveness in achieving weight- or muscle-related goals. Future research should examine how these subjective experiences shape subsequent supplement purchases, risk perceptions, and decision-making processes, as such insights may support more reflective and health-informed choices among adolescents.

Despite these limitations, this study provides novel evidence linking online health information-seeking and the internalization of appearance ideals with adolescents' purchasing of weight-loss and muscle-building products. Future research could explore evolving appearance norms in digital contexts, their internalization among adolescents with diverse gender identities and sexual orientations, as well as additional media-related variables (e.g., e-health literacy, trust in online health information) that may further shape supplement purchasing behaviors.

### 4.2 Conclusion

This study demonstrates that adolescents' internalization of appearance ideals and online engagement with weight- and fitness-related information are associated with supplement consumerism, revealing a potential pathway through which online media may influence youth health behaviors. From a public health perspective, these findings underscore the need to monitor online health behaviors, regulate supplement marketing, implement evidence-based prevention strategies such as media literacy and adolescent-focused health education, and further investigate the long-term consequences of adolescent supplement consumerism.

## Data Availability

The datasets presented in this study can be found in online repositories. The names of the repository/repositories and accession number(s) can be found below: The data and the analyses scripts are shared via the Open Science Framework: https://osf.io/a3rft/?view_only=605f8677b11747caa1a91abf0a7784c3.

## References

[B1] Almenara C. A. Gulec H. (2024). Uncovering the top nonadvertising weight loss websites on Google: a data-mining approach. JMIR Infodemiology 4:e51701. doi: 10.2196/51701 39661980 PMC11669867

[B2] Alves C. Lima R. V. (2009). Dietary supplement use by adolescents. J. Pediatr. 85, 287–294. doi: 10.1590/S0021-75572009000400004 19585056

[B3] Austin S. B. Liu S. H. Tefft N. (2018). Could a tax on unhealthy products sold for weight loss reduce consumer use? A novel estimation of potential taxation effects. Prev. Med. 114, 39–46. doi: 10.1016/j.ypmed.2018.05.022 29842920

[B4] Austin S. B. Yu K. Tran A. Mayer B. (2017). Research-to-policy translation for prevention of disordered weight and shape control behaviors: a case example targeting dietary supplements sold for weight loss and muscle building. Eat. Behav. 25, 9–14. doi: 10.1016/j.eatbeh.2016.03.037 27118415

[B5] Austin S. B. Ziyadeh N. Kahn J. A. Camargo Jr C. A. Colditz G. A. Field A. E. (2004). Sexual orientation, weight concerns, and eating-disordered behaviors in adolescent girls and boys. J. Am. Acad. Child Adolesc. Psychiatry 43, 1115–1123. doi: 10.1097/01.chi.0000131139.93862.10 15322415

[B6] Avelar-Escobar G. Méndez-Navarro J. Ortiz-Olvera N. X. Castellanos G. Ramos R. Gallardo-Cabrera V. E. . (2012). Hepatotoxicity associated with dietary energy supplements: use and abuse by young athletes. Ann. Hepatol. 11, 564–569. doi: 10.1016/S1665-2681(19)31474-7 22700641

[B7] Barretto J. R. Gouveia M. Alves C. (2024). Use of dietary supplements by children and adolescents. J. Pediatr. 100, S31–S39. doi: 10.1016/j.jped.2023.09.008 38529679 PMC10960193

[B8] Bell A. Dorsch K. D. McCreary D. R. Hovey R. (2004). A look at nutritional supplement use in adolescents. J. Adolesc. Health 34, 508–516. doi: 10.1016/S1054-139X(03)00348-3 15145408

[B9] Biefeld S. D. Mays K. Brown C. S. (2025). “Thin and waif-like”: body image and body ideals in gender-expansive adults. J. Gend. Stud. 1–22. doi: 10.1080/09589236.2025.2552808

[B10] Boutelle K. Neumark-Sztainer D. Story M. Resnick M. (2002). Weight control behaviors among obese, overweight, and nonoverweight adolescents. J. Pediatr. Psychol. 27, 531–540. doi: 10.1093/jpepsy/27.6.531 12177253

[B11] Bozsik F. Whisenhunt B. L. Hudson D. L. Bennett B. Lundgren J. D. (2018). Thin is in? Think again: the rising importance of muscularity in the thin ideal female body. Sex Roles 79, 609–615. doi: 10.1007/s11199-017-0886-0

[B12] Buchanan L. Kelly B. Yeatman H. Kariippanon K. (2018). The effects of digital marketing of unhealthy commodities on young people: a systematic review. Nutrients 10:148. doi: 10.3390/nu10020148 29382140 PMC5852724

[B13] Calzo J. P. Masyn K. E. Corliss H. L. Scherer E. A. Field A. E. Austin S. B. (2015). Patterns of body image concerns and disordered weight- and shape-related behaviors in heterosexual and sexual minority adolescent males. Dev. Psychol. 51, 1216–1225. doi: 10.1037/dev0000027 26098578 PMC4540685

[B14] Cusumano D. L. Thompson J. K. (2001). Media influence and body image in 8-11-year-old boys and girls: a preliminary report on the multidimensional media influence scale. Int. J. Eat. Disord. 29, 37–44. doi: 10.1002/1098-108x(200101)29:1<37::aid-eat6>3.0.co;2-g 11135331

[B15] Engel E. Gell S. Heiss R. Karsay K. (2024). Social media influencers and adolescents' health: a scoping review of the research field. Soc. Sci. Med. 340:116387. doi: 10.1016/j.socscimed.2023.116387 38039770

[B16] Field A. E. Austin S. B. Camargo Jr C. A. Taylor C. B. Striegel-Moore R. H. Loud K. J. . (2005). Exposure to the mass media, body shape concerns, and use of supplements to improve weight and shape among male and female adolescents. Pediatrics 116, e214–e220. doi: 10.1542/peds.2004-2022 16061574

[B17] Frison E. Vandenbosch L. Eggermont S. (2013). Exposure to media predicts use of dietary supplements and anabolic-androgenic steroids among Flemish adolescent boys. Eur. J. Pediatr. 172, 1387–1392. doi: 10.1007/s00431-013-2056-x 23748985

[B18] Ganson K. T. Nguyen L. Ali A. R. H. Hallward L. Jackson D. B. Testa A. . (2023). Associations between social media use, fitness- and weight-related online content, and use of legal appearance- and performance-enhancing drugs and substances. Eat. Behav. 49:101736. doi: 10.1016/j.eatbeh.2023.101736 37141803

[B19] Ganson K. T. Pang N. Testa A. Murray S. B. Nagata J. M. (2024). Describing use of muscle-building supplements among adolescents and young adults in Canada. Perform. Enhanc. Health 12:100284. doi: 10.1016/j.peh.2024.100284

[B20] Garner D. M. (2004). EDI-3: Eating Disorder Inventory-3: Professional Manual. Lutz, FL: Psychological Assessment Resources.

[B21] Greškovičová K. Masaryk R. Synak N. Cavojová V. (2022). Superlatives, clickbaits, appeals to authority, poor grammar, or boldface: is editorial style related to the credibility of online health messages? Front. Psychol. 13:940903. doi: 10.3389/fpsyg.2022.940903 36106046 PMC9465483

[B22] Helfert S. Warschburger P. (2011). A prospective study on the impact of peer and parental pressure on body dissatisfaction in adolescent girls and boys. Body Image 8, 101–109. doi: 10.1016/j.bodyim.2011.01.004 21354379

[B23] Henriksen H. A. Rozzell-Voss K. N. Pennesi J.-L. Askew A. J. Convertino A. D. Blashill A. J. (2025). Muscularity-oriented attitudes and behaviors among college-aged women: the role of sexual orientation. Body Image 52:101822. doi: 10.1016/j.bodyim.2024.101822 39673976

[B24] Hildebrandt T. Harty S. Langenbucher J. W. (2012). Fitness supplements as a gateway substance for anabolic-androgenic steroid use. Psychol. Addict. Behav. 26, 955–962. doi: 10.1037/a0027877 22486333 PMC3838896

[B25] Hoffman J. R. Faigenbaum A. D. Ratamess N. A. Ross R. Kang J. Tenenbaum G. (2008). Nutritional supplementation and anabolic steroid use in adolescents. Med. Sci. Sports Exerc. 40, 15–24. doi: 10.1249/mss.0b013e31815a5181 18091024

[B26] IBM Corp (2021). IBM SPSS Statistics for Windows (Version 28.0). Armonk, NY: IBM Corp.

[B27] Kvardova N. Lacko D. Machackova H. (2023). The validity of the Czech version of Body Appreciation Scale-2 for adolescents. J. Eat. Disord. 11:176. doi: 10.1186/s40337-023-00897-7 37798665 PMC10557209

[B28] Lacko D. Machackova H. Slavík L. (2024). Adolescents' perceptions of the credibility of informational content on fitness and dietary supplements: the impact of banner and native advertising. J. Adolesc. 96, 1956–1968. doi: 10.1002/jad.12394 39164994 PMC11618704

[B29] Lev Arey D. Peleg Y. Gutman T. (2025). Male body image in focus: muscularity-oriented eating behaviours, muscle dysmorphia, and exercise addiction in gay and heterosexual men. J. Eat. Disord. 13:151. doi: 10.1186/s40337-025-01311-0 40696468 PMC12285041

[B30] Levine M. P. Murnen S. K. (2009). “Everybody knows that mass media are/are not [pick one] a cause of eating disorders”: a critical review. J. Soc. Clin. Psychol. 28, 9–42. doi: 10.1521/jscp.2009.28.1.9

[B31] Liechty J. M. Lee M. J. (2013). Longitudinal predictors of dieting and disordered eating among young adults in the U.S. Int. J. Eat. Disord. 46, 790–800. doi: 10.1002/eat.22174 23983018

[B32] McCabe M. P. Ricciardelli L. A. (2009). Extreme weight change behaviours: are overweight and normal weight adolescents different? Eur. Eat. Disord. Rev. 17, 301–314. doi: 10.1002/erv.929 19378347

[B33] Nagata J. M. Braudt D. B. Domingue B. W. Bibbins-Domingo K. Garber A. K. Griffiths S. . (2019). Genetic risk, body mass index, and weight control behaviors. Int. J. Eat. Disord. 52, 825–833. doi: 10.1002/eat.23083 30994932 PMC6609475

[B34] Nagata J. M. Domingue B. W. Darmstadt G. L. Weber A. M. Meausoone V. Cislaghi B. . (2020). Gender norms and weight control behaviors in U.S. adolescents. J. Adolesc. Health 66, S34–S41. doi: 10.1016/j.jadohealth.2019.08.020 31866036 PMC6928570

[B35] Neumark-Sztainer D. Paxton S. J. Hannan P. J. Haines J. Story M. (2006). Does body satisfaction matter? Five-year longitudinal associations. J. Adolesc. Health 39, 244–251. doi: 10.1016/j.jadohealth.2005.12.001 16857537

[B36] O'Dea J. A. (2003). Consumption of nutritional supplements among adolescents. Health Educ. Res. 18, 98–107. doi: 10.1093/her/18.1.98 12608687

[B37] Picciano M. F. Dwyer J. T. Radimer K. L. Wilson D. H. Fisher K. D. Thomas P. R. . (2007). Dietary supplement use among infants, children, and adolescents. Arch. Pediatr. Adolesc. Med. 161, 978–985. doi: 10.1001/archpedi.161.10.978 17909142

[B38] Pomeranz J. L. Barbosa G. Killian C. Austin S. B. (2015). The dangerous mix of adolescents and dietary supplements. J. Public Health Manag. Pract. 21, 496–503. doi: 10.1097/PHH.0000000000000142 25248073

[B39] Rideout V. Fox S. Peebles A. Robb M. B. (2021). Coping With COVID-19: How Young People Use Digital Media to Manage Their Mental Health. Available online at: https://www.commonsensemedia.org/ (Accessed October 1, 2025).

[B40] Rideout V. Fox S. Well Being Trust (2018). Digital Health Practices, Social Media Use, and Mental Well-Being Among Teens and Young Adults in the U.S. Articles, Abstracts, and Reports, 1093. Available online at: https://digitalcommons.providence.org/publications/1093 (Accessed October 1, 2025).

[B41] Roberts S. R. Maheux A. J. Hunt R. A. Ladd B. A. Choukas-Bradley S. (2022). Incorporating social media and muscular ideal internalization. Body Image 41, 239–247. doi: 10.1016/j.bodyim.2022.03.002 35306356

[B42] Rocha T. Amaral J. S. Oliveira M. (2016). Adulteration of dietary supplements by the illegal addition of synthetic drugs: a review. Compr. Rev. Food Sci. Food Saf. 15, 43–62. doi: 10.1111/1541-4337.12173 33371574

[B43] Rodgers R. F. Slater A. Gordon C. S. McLean S. A. Jarman H. K. Paxton S. J. (2020). A biopsychosocial model of social media use. J. Youth Adolesc. 49, 399–409. doi: 10.1007/s10964-019-01190-0 31907699

[B44] Ruano J. Teixeira V. H. (2020). Prevalence of dietary supplement use by gym members. J. Int. Soc. Sports Nutr. 17:11. doi: 10.1186/s12970-020-00342-z 32093724 PMC7038552

[B45] Ruggiero T. E. (2000). Uses and gratifications theory in the 21st century. Mass Commun. Soc. 3, 3–37. doi: 10.1207/S15327825MCS0301_02

[B46] Saraipour F. Panahi S. Nemati-Anaraki L. Shahraki-Mohammadi A. (2025). Adolescents' health information–seeking behavior. J. Adolesc. Health 77, 592–601. doi: 10.1016/j.jadohealth.2025.05.030 40767803

[B47] Subrahmanyam K. Šmahel D. (2011). Digital Youth: The Role of Media in Development. New York, NY: Springer Science + Business Media.

[B48] Svantorp-Tveiten K. M. E. Friborg O. Torstveit M. K. Mathisen T. F. Sundgot-Borgen C. Rosenvinge J. H. . (2021). Protein, creatine, and dieting supplements among adolescents. Front. Sports Act. Living 3:727372. doi: 10.3389/fspor.2021.727372 34723179 PMC8548763

[B49] Thompson J. K. Coovert M. D. Stormer S. M. (1999a). Body image, social comparison, and eating disturbance. Int. J. Eat. Disord. 26, 43–51. doi: 10.1002/(sici)1098-108x(199907)26:1<43::aid-eat6>3.0.co;2-r 10349583

[B50] Thompson J. K. Heinberg L. J. Altabe M. Tantleff-Dunn S. (1999b). Exacting Beauty: Theory, Assessment, and Treatment of Body Image Disturbance. Washington, DC: APA. doi: 10.1037/10312-000

[B51] Tiggemann M. Slater A. (2014). NetTweens: The Internet and Body Image Concerns. London: Sage Publications.

[B52] van den Berg P. Thompson J. K. Obremski-Brandon K. Coovert M. (2002). The tripartite influence model. J. Psychosom. Res. 53, 1007–1020. doi: 10.1016/S0022-3999(02)00499-3 12445590

[B53] Vankerckhoven L. Raemen L. Claes L. Eggermont S. Palmeroni N. Luyckx K. (2023). Identity formation, body image, and body-related symptoms. J. Youth Adolesc. 52, 651–669. doi: 10.1007/s10964-022-01717-y 36484894 PMC9735114

[B54] Wartella E. Rideout V. Montague H. Beaudoin-Ryan L. Lauricella A. (2016). Teens, health and technology. Media Commun. 4, 13–23. doi: 10.17645/mac.v4i3.515

[B55] Yanover T. Thompson J. K. (2010). Perceptions of health and attractiveness: the effects of body fat, muscularity, gender, and ethnicity. J. Health Psychol. 15, 1039–1048. doi: 10.1177/1359105309360426 20511287

[B56] Yergaliyev K. A. Aveling E.-L. Lee R. M. Austin S. B. (2020). Lessons for policy initiatives. J. Adolesc. Health 67, 550–556. doi: 10.1016/j.jadohealth.2020.03.026 32387096

